# NADPH oxidase 1: A target in the capacity of dimeric ECG and EGCG procyanidins to inhibit colorectal cancer cell invasion

**DOI:** 10.1016/j.redox.2023.102827

**Published:** 2023-07-25

**Authors:** Wei Zhu, Patricia I. Oteiza

**Affiliations:** a Department of Nutrition, University of California, Davis, CA, USA; b Department of Environmental Toxicology, University of California, Davis, CA, USA

**Keywords:** Colorectal cancer, Procyanidins, Epidermal growth factor receptor, NADPH oxidase 1, Matrix metalloproteinases, Cancer

## Abstract

Colorectal cancer (CRC) is prevalent worldwide. Dietary consumption of procyanidins has been linked to a reduced risk of developing CRC. The epidermal growth factor (EGF) receptor (EGFR) signaling pathway is frequently dysregulated in CRC. Our earlier research showed that the procyanidin dimers of epicatechin gallate (ECG) and epigallocatechin gallate (EGCG), through their interaction with lipid rafts, inhibit the EGFR signaling pathway and decrease CRC cell growth. The process of cancer cell invasion and metastasis involves matrix metalloproteinases (MMPs), which are partially EGFR-regulated. This study investigated whether ECG and EGCG dimers can inhibit EGF-induced CRC cell invasion by suppressing the redox-regulated activation of the EGFR/MMPs pathway. Both dimers mitigated EGF-induced cell invasion and the associated increase of MMP-2/9 expression and activity in different CRC cell lines. In Caco-2 cells, both dimers inhibited the activation of the EGFR and downstream of NF-κB, ERK1/2 and Akt, which was associated with decreased MMP-2/9 transcription. EGF induced a rapid NOX1-dependent oxidant increase, which was diminished by both ECG and EGCG dimers and NOX inhibitors (apocynin, Vas-2870, DPI). Both dimers inhibited NOX1 gene expression, as well as NOX1 activity with evidence of direct binding to NOX1. Both dimers, all NOX chemical inhibitors and NOX1 silencing inhibited EGF-mediated activation of the EGFR signaling pathway and the increased MMP-2/9 mRNA levels and activity. Pointing to the relevance of NOX1 on ECG and EGCG dimer effects on CRC invasiveness, silencing of NOX1 also inhibited EGF-stimulated Caco-2 cell invasion. In summary, ECG and EGCG dimers can act inhibiting CRC cell invasion/metastasis both, by downregulating MMP-2 and MMP-9 expression via a NOX1/EGFR-dependent mechanism, and through a direct inhibitory effect on MMPs enzyme activity.

## Highlights

• Dimeric procyanidins inhibit epidermal growth factor (EGF)-induced colorectal cancer cell (CRC) invasion.
• Dimers acts inhibiting EGF receptor (EGFR) signaling and matrix metalloproteases (MMP-2/9) expression and activity.
• NOX1 is required for EGFR-mediated MMP-2/9 expression, and cell invasion.
• Dimers inhibit EGF-induced CRC invasion by downregulating NOX1 and MMP-2/9.

## Graphical abstract

**Figure:**
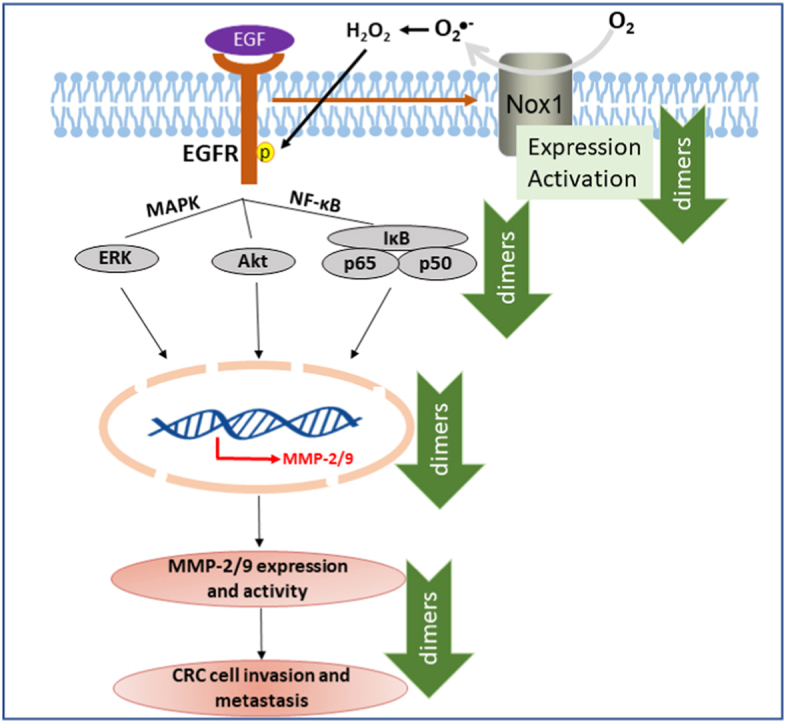

## 1 Introduction

In the United States, colorectal cancer (CRC) is the third most frequently diagnosed cancer and the second leading cause of cancer-associated deaths [1]. Globally, CRC constitutes 10% of the total cancer cases and 9.4% of all cancer-associated deaths. Additionally, it is projected that in 2040 new cases of CRC will increase to 3.2 million worldwide [2]. Despite the effectiveness of surgery as a curative treatment for CRC, the risk of recurrence and metastasis remains still high. Although the incidence of CRC has been reduced and the prognosis improved through the development of new therapeutic approaches and early detection tests, around 50% of CRC cases become metastatic and 40% of patients ultimately die due to CRC metastasis [3,4].

The critical event in the process of CRC invasion and metastasis is the degradation of the extracellular matrix (ECM) surrounding the tumor tissue. Matrix metalloproteases (MMPs), a family of zinc- and calcium-dependent proteolytic enzymes, facilitate cancer cell invasion and dissemination by degrading the basement membrane and ECM proteins [[5], [6], [7]]. Dysregulation of MMPs is now regarded as an early contributing mechanism for cancer initiation and progression. Among MMPs, MMP-2 and MMP-9 are correlated with CRC disease stage and/or prognosis [[8], [9], [10]]. Thus, high expression of MMP-2/9 predicts a poor CRC survival outcome [11]. Furthermore, MMP-9 is regarded as a novel biomarker and potential therapeutic target in many human cancers [[12], [13], [14], [15]]. Overall, the inhibition of MMP-2/9 expression and/or activity emerge as a potential strategy against CRC metastasis.

NADPH oxidase (NOX) 1 (NOX1) is the main NOX isoform in intestinal epithelial cells. In the gastrointestinal (GI) tract, NOX1 participates in the regulation of the GI innate immune response [16], in stem and CRC cell proliferation [17,18] and CRC cell migration and metastasis [19,20]. NOX1 is activated when the epidermal growth factor (EGF) binds to its receptor (EGFR) which causes transient O_2_^.-^/H_2_O_2_ increases leading to events, i.e. oxidation of protein tyrosine phosphatase (PTP) and EGFR cysteine residues, which in turn prolong the EGFR signaling cascade [21,22]. Overactivation of the EGFR and downstream cascades, including MEK/ERK and PI3K/Akt, is frequent in cancer [23,24]. NOX1 activation, increased oxidant production and EGFR activation can lead to the upregulation of MMPs which can then promote tumor metastasis [20,25,26]. Thus, dietary strategies that inhibit NOX1/EGFR-mediating signaling could play a key role in mitigating CRC metastasis.

**Fig. 1:**
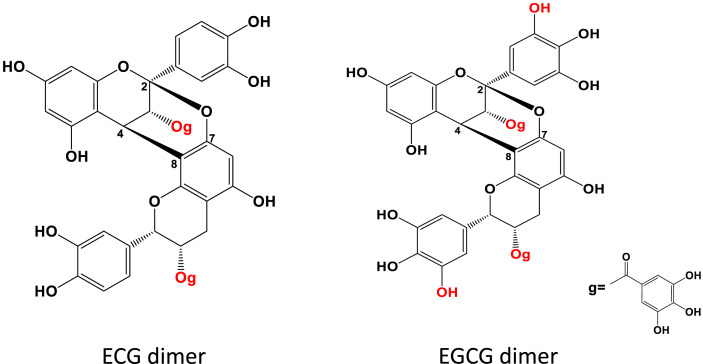
**Chemical structures of epicatechin-3-gallate (ECG) and epigallocatechin-3-gallate (EGCG) dimers.** Dimers are linked by 4β→8 and 2β→O→7 bonds. g: galloyl moiety.

Diet has a major influence on CRC risk since intestinal epithelial cells are directly exposed to large amounts of dietary components [27,28], making the search for dietary bioactives that can mitigate CRC development and metastasis highly relevant [29]. Evidence from epidemiological and clinical studies suggest that diets rich in fruit and vegetables can reduce the risk of developing CRC [[30], [31], [32], [33], [34]]. Procyanidins (PCA), which are abundant in fruit and vegetables, have beneficial health effects at the GI tract [35,36], and their consumption is related to a reduced risk of CRC in humans [37,38]. We previously observed that two dimeric PCA isolated from persimmon fruits, composed of epicatechin-3-gallate (ECG) and epigallocatechin-3-gallate (EGCG) subunits linked by 4β→8 and 2β→O→7 bonds (Fig. 1), inhibit *in vitro* CRC cell growth and promote apoptosis by regulating the EGFR pathway [39]. Considering, the NOX-dependent redox regulation of EGFR activation in tumor progression [21,22,40], and previous evidence showing that PCA modulate oncogenic signals, i.e. ERK1/2, Akt, NF-κB, in part by mitigating NOX-mediated transient O_2_^.-^/H_2_O_2_ increases [21,41,42], the present study investigated if ECG and EGCG dimers could inhibit CRC cell invasion through a EGFR/NOX1-dependent MMP-2/9 downregulation. In CRC cell lines, we assessed the capacity of ECG and EGCG dimers to: i) inhibit EGF-stimulated MMP-2/9 expression/activity and cell invasion, ii) inhibit EGF-stimulated signaling cascades that promote MMP-2/9 transcription, i.e. NF-κB (IKKα, p65), ERK1/2 and Akt; iii) inhibit O_2_^.-^/H_2_O_2_ production through NOX modulation (expression/activity). The role of NOX1/EGFR in regulating Caco-2 cell invasion through MMP-2/9 upregulation was further assessed through NOX1 silencing. Results show that ECG and EGCG dimers inhibition and suppression of NOX1 is the mechanism largely involved in their capacity to inhibit EGF-mediated cell invasion.

## 2 Materials and methods

### 2.1 Materials

Human CRC cell lines HT29, SW480, HCT15, HCT116 and Caco-2 were all purchased from the American Type Culture Collection (ATCC). Cell culture media McCoy's 5A, RPMI, DMEM, MEM and Opti-MEM, fetal bovine serum (FBS) and antibiotics penicillin-streptomycin were from Gibco (Waltham, MA). EGF was obtained from PeproTech (Rocky Hill, NJ). Dihydroethidium (DHE) was from EMD Millipore (Hayward, CA), and 5-(and-6)-carboxy-2’,7’-dichlorodihydrofluorescein diacetate (DHDCF) and Amplex Red Hydrogen Peroxide/Peroxidase Assay Kit, RIPA buffer, TRIzol reagent, lipofectamine RNAiMAX reagent, scramble siRNA and NOX1 siRNA were from Invitrogen/Life Technologies (Grand Island, NY). Apocynin (Apo), diphenyleneiodonium (DPI), VAS-2870 (Vas), sulphorhodamine B (SRB) and porcine gelatin were from Sigma-Aldrich (St. Louis, MO). PVDF membranes and ECL reagent for Western blot were from Bio-Rad (Hercules, CA). Reagents for the electrophoretic mobility shift assay (EMSA) were obtained from Santa Cruz Biotechnology (Santa Cruz, CA) and Promega (Madison, WI). Primary antibodies for p(Tyr1068)-EGFR (#3777), EGFR (#4267), p(Ser176/180)-IKKα/β (2697), IKKα (#2682), p(Ser536)-p65 (#3033), p65 (#8242), p(Ser473)-Akt (#4060), Akt (#4691), p(Thr202, Tyr204)-ERK (#4370), ERK (#9102) and β-actin (#12620); secondary antibodies anti-rabbit HRP-conjugated (#7074), anti-rabbit biotinylated (#14708), streptavidin (#3999) and the biotinylated ladder (#7727) were from Cell Signaling Technology, Inc. (Danvers, MA). The primary antibody for NOX1 (#ab78016) was from Abcam (Cambridge, England).

### 2.2 Methods

#### 2.2.1 Isolation and purification of ECG and EGCG dimers

ECG and EGCG dimers were obtained from persimmon fruits as described before with minor modifications [43]. After AB-8 resin purification, a medium-pressure liquid chromatography was applied first to purify the extracts before being loaded to a preparative liquid chromatography for ECG and EGCG dimers purification and collection [39]. The purity was over 95% for both dimers using procyanidin A2 as a standard. The structures of ECG and EGCG dimers are shown in Fig. 1.

#### 2.2.2 Cell culture and treatments

Caco-2 cells were cultured in MEM medium while HT29 and HCT116 cells were cultured in McCoy’s 5A medium. RMPI media was used for the culture of SW480 and HCT15 cells. All media were supplemented with 10% (v/v) FBS, 1% (v/v) NEAA, 1% (v/v) sodium pyruvate and 0.5% (v/v) penicillin-streptomycin during cell growth. Cells were split when they reached 70–80% confluency. For the experiments, cells were seeded and allowed to grow for 36–48 h before being starved for 12 h in FBS-free MEM. Subsequently, cells were preincubated for 30 min with or without ECG or EGCG dimers at the concentrations described for each experiment, followed by incubation without or with 10 ng/ml EGF for 10 min-6 h.

#### 2.2.3 Western blot

Following the corresponding treatments, total cell homogenates were prepared as previously described [41]. Protein concentration was measured using the Bradford assay [44] and an aliquot of protein (30–50 μg) was mixed with 4X sample buffer before being separated on a 7% (w/v) SDS-PAGE gel and transferred onto PVDF membranes. Two different molecular weight standards (Colored and biotinylated) were loaded onto the gels simultaneously. Following transfer, membranes were blocked with 5% (w/v) non-fat milk for 1 h, followed by overnight incubation in the presence of the corresponding antibodies (1:1,000 dilution) in 1% (w/v) bovine serum albumin in TBS buffer (50 mM Tris, 150 mM NaCl, 0.1% (v/v) Tween-20, pH = 7.8). After 90 min incubation at room temperature in the presence of the HRP-conjugated secondary antibody (1:10,000), the bands were visualized by chemiluminescence detection in a ChemiDoc Imaging System (Bio-Rad, Hercules, CA) and quantified using Image lab (Bio-Rad Laboratories, Hercules, CA).

#### 2.2.4 Quantitative polymerase chain reaction (qPCR)

For qPCR determinations, RNA was extracted from proliferating Caco-2 cells after treatments. Reverse transcription was done to generate cDNA using the high-capacity cDNA Reverse Transcriptase kit (Applied Biosystems, Grand Island, NY). mRNA levels of NOX1, MMP-2, MMP-9 and β-actin were assessed by qPCR (iCycler, Bio-Rad, Hercules, CA). β-actin was used as the housekeeping gene. The relative fold change in mRNA levels of each gene was calculated using the 2^−ΔΔCt^ method [45]. The primers used in present study were as follows:

NOX1 forward: 5′-GTACAAATTCCAGTGTGCAGACCAC-3′

NOX1 reverse: 5’-CAGACTGGAATATCGGTGACAGCA-3’;

MMP-2 forward: 5’-AGCGAGTGGATGCCGCCTTTAA-3’

MMP-2 reverse: 5’-CATTCCAGGCATCTGCGATGAG-3’

MMP-9 forward: 5’-GATGCGTGGAGAGTCGAAAT-3’

MMP-9 reverse: 5’-CACCAAACTGGATGACGATG-3’

β-actin forward: 5′-TCATGAAGTGTGACGTGGACATCCGC-3′

β-actin reverse: 5′-CCTAGAAGCATTTGCGGTGCACGATG-3′

For the evaluation of MMP-2, MMP-9 and NOX1 mRNA stability, gene transcription was inhibited with Actinomycin D [46]. 1 × 10^6^ cells were seeded in 60 mm^2^ dishes and allowed to grow for 48 h. After 12 h starvation, cells were treated with/without EGF and dimers for 6 h. For the first-time point (t = 0), a subset of cells were collected with TRIzol. The rest of the dishes were added with Actinomycin D to a final concentration of 10 μg/ml. Cells were collected at 0.5, 1, 2, 4 and 6 h following Actinomycin D addition. RNA isolation, reverse transcription to cDNA and qPCR were done as described above. The Ct average value at each time point was normalized to the Ct average value at t = 0 to calculate ΔCt values for the control and treatment groups (ΔCt = (Average Ct of each time point - Average Ct of t = 0)). To calculate the relative abundance at each time point we used 2^-ΔCT^.

#### 2.2.5 Electrophoretic mobility shift assay (EMSA)

EMSA is a highly sensitive approach for detecting protein-nucleic acid interactions. After the corresponding treatments, nuclear fractions were isolated as previously described with minor modifications [42]. The isolated nuclear fractions were incubated with the labeled oligonucleotide (20,000–30,000 cpm) in 1X binding buffer (10 mM Tris–HCl buffer, containing 4% (v/v) glycerol, 1 mM MgCl_2_, 0.5 mM EDTA, 0.5 mM dithiothreitol, 50 mM NaCl, and 0.05 mg/ml poly(dI–dC), pH = 7.5) for 20 min at room temperature. The products were then separated by electrophoresis in a 5% (w/v) nondenaturing polyacrylamide gel with 0.5X TBE (45 mM Tris–borate, 1 mM EDTA). Gels were subsequently dried, and the level of radioactivity was measured using a Phosphoimager 840 (Amersham Pharmacia Biotech).

#### 2.2.6 Determination of cell oxidant levels

Cell oxidant levels were assessed using the probes DCFDA, DHE and Amplex Red as previously described [21]. Caco-2 cells were seeded in 96-well plates at a 5 × 10^4^ cells/well density. After reaching 70–80% confluency, cells were starved in FBS-free MEM for 12 h. Cells were then preincubated with or without 1x IC_50_ ECG or EGCG dimer (IC_50_ values were from our previous report of the Caco-2 cancer cell variability inhibition by the ECG and EGCG dimers [39]), or NOX inhibitors (1 μM Apo, 1 μM Vas or 1 μM DPI) for 30 min, and subsequently incubated for 10 min with or without 10 ng/ml EGF. Then, the medium was removed, and cells added with 20 μM DHE or 25 μM DCF and incubated for 30 min at 37 °C. The medium was removed, cells washed with PBS twice and fluorescence was measured in 100 μl PBS in a microplate reader (Bio-Tek, Winooski, VT) at λexc: 485 nm; λexc: 535 nm for oxidized DCFDA, and at λexc: 485 nm; λexc: 535 nm for oxidized DHE. H_2_O_2_ released to the medium was measured after 10 min EGF addition using the Hydrogen Peroxide/Peroxidase Assay Kit and following the manufacturer's protocol. The fluorescence for all probes was normalized to the protein content using SRB [47].

#### 2.2.7 Gelatin zymography

The activity of MMP-2/9 was analyzed by gelatin zymography according to a previous report [48]. 1 × 10^6^ cells proliferating Caco-2 cells were seeded in 60 mm^2^ dishes, grown to 70–80% confluency and starved in serum-free MEM for 12 h. Cells were then preincubated with or without 5–60 μM ECG or EGCG dimer, or NOX inhibitors (1 μM Apo, 1 μM Vas or 1 μM DPI) for 30 min, and subsequently incubated for 6 h with or without 10 ng/ml EGF. All media were collected, centrifuged at 800×*g* for 8 min to remove cell debris, and the supernatant was concentrated with a Vacufuge concentrator (Eppendorf, Germany). The concentrated samples were loaded on 7% gelatin-containing SDS-PAGE gels. Gels were washed 3 times and then subsequently pre-equilibrated with fresh incubation buffer (50 mM Tris-HCl, 5 mM CaCl_2_, 1 μM ZnCl_2_, 1% (v/v) Triton X-100, pH = 7.5) for 10 min at 37 °C. Gels were further incubated with fresh incubation buffer for 24 h at 37 °C. After being washed 3 times, gels were stained with Coomassie Brilliant Blue R-250 (Bio-Rad Laboratories, Hercules, CA) for 0.5–1 h, and rinsed 3 times with a destaining solution containing 40% (v/v) methanol and 10% (v/v) acetic acid, until clear white bands were seen due to gelatinase activity. Bands were visualized in a ChemiDoc (Bio-Rad Laboratories, Hercules, CA), and were quantified by densitometry using the Image Lab (Bio-Rad Laboratories, Hercules, CA).

#### 2.2.8 NOX1 silencing

Transfections were carried out using lipofectamine following the manufacturer's protocol. Caco-2 cells in log-phase were plated into 6-well plates (0.2 × 10^6^ cells/well) in 2 ml growth medium and incubated for 24 h to reach 30–50% confluency at the time of transfection. Cells were transfected with 30 pmol NOX1 siRNA and incubated in complete media for 24, 48 or 72 h, respectively. Cells were collected and processed to evaluate NOX1 mRNA or protein levels as described above. For the treatments, cells were used after 48 h of initiating the silencing.

#### 2.2.9 Cell invasion assay

The cell invasion assay was performed using Matrigel-coated transwell cell culture chambers (Corning, MA). Normal or siRNA transfected proliferating Caco-2 cells were collected and resuspended at a density of 1 × 10^5^ cells/ml in FBS-free media. 250 μl of cell suspension were added to the upper chamber and 500 μl of 10% (v/v) FBS in MEM medium added to the lower chamber. Cells were incubated first with or without ECG or EGCG dimers for 30 min, and subsequently with or without EGF (10 ng/ml) for 48 h. All additions were made to the upper chamber. Subsequently, the medium in the upper chamber was removed, transwell inserts were washed twice with PBS, cells fixed with paraformaldehyde (3.7% (w/v) in PBS) for 2 min and permeabilized in 100% methanol for 20 min. Cells in the upper chamber were removed using a cotton swab and the transwell chambers air dried. Invasive cells migrating to the back of the membrane were stained with 0.1% (w/v in PBS) crystal violet. The invasiveness of Caco-2 cells was defined as the total number of cells in 3 randomly selected microscopic fields.

#### 2.2.10 Statistical analysis

All experiments were performed at 3–7 independent experiments and results are shown as means ± SEM. Statistical analysis were performed using GraphPad Prism 8.0 software (IBM Inc., Armonk, NY). Data were tested for normal Gaussian curve (bell-shape) distribution using the Shapiro-Wilk test. In case of normal distribution, equal variances were tested using Bartlett’s test and subsequently data were analyzed using one-way ANOVA followed by Fisher least significance difference test to evaluate significant differences among the groups’ treatments. Outliers were identified using the Inter Quartile Range (IQR) test. *P* < 0.05 was considered statistically significant.

## 3 Results

### 3.1 ECG and EGCG dimers inhibited EGF-mediated cell invasion, MMP-2/9 activation and MMP-2/9 increased mRNA levels in Caco-2 cells

**Fig. 2:**
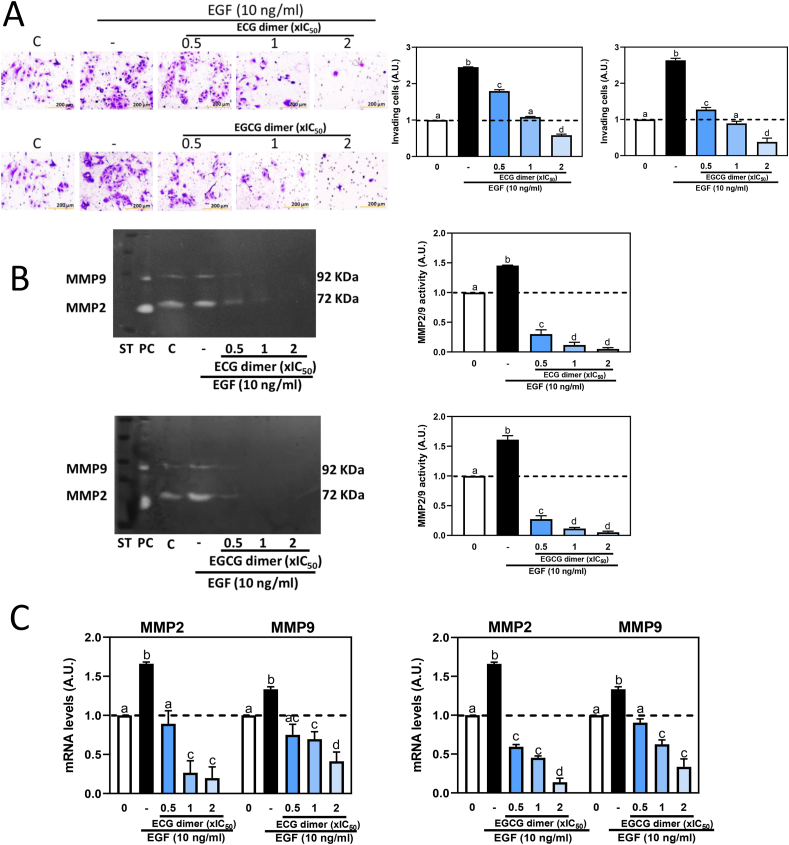
**ECG and EGCG dimers inhibited EGF-mediated cell invasion, MMP2/9 activation and MMP-2/9 increased mRNA levels in Caco-2 cells**. Cells were pre-incubated with or without ECG and EGCG dimers for 30 min and then with or without EGF (10 ng/ml) for 24 h for the assessment of cell invasion or 6 h for the determination of MMP-2/9 activity and mRNA levels. The concentrations of ECG and EGCG dimers used corresponds to 0.5x, 1x and 2x IC_50_ previously reported for the effects of the dimers on Caco-2 cell viability [39]. A- Cell invasion (20X magnification), B- MMP-2/9 activity in the cell culture medium and C- MMP-2/9 mRNA levels were assessed as described in methods. MMP-2/9 mRNA levels measured by qPCR were referred to actin mRNA levels as housekeeping gene. Results are shown as means ± SEM of 3–5 independent experiments. Values having different superscripts are significantly different (p < 0.05, One-way ANOVA-test).

Based on the IC_50_ values that we previously reported for the inhibitory effects of the dimers on Caco-2 cell growth [39], we currently used 0.5x, 1x and 2x IC_50_ concentrations (5, 10, 20 μM for ECG dimer and 15, 30, 60 μM for EGCG dimer) to evaluate the effects of dimers on CRC cell invasion and the underlying mechanisms. We first examined the effects of ECG and EGCG dimers on EGF-induced cell invasion. The number of invading cells was markedly increased by EGF (approximately 2.5 folds compared to control, non-added cells) (Fig. 2A). Treatment with both dimers dose-dependently inhibited EGF-induced cell invasiveness.

MMP-2 and MMP-9 are the predominant enzymes found overexpressed in metastatic cancers. They facilitate ECM degradation, being associated with primary tumor growth, invasion and metastasis [49]. Zymographic analysis, based on the capacity of MMP-2 and MMP-9 to degrade gelatin, showed that EGF caused an approximate 1.5-fold increase in MMP-2/9 activity, while ECG and EGCG dimers almost completely inhibited MMP-2/9 activity in the range of concentrations tested (Fig. 2B). The effect of EGF and the dimers on MMP-2 and MMP-9 mRNA levels was next evaluated. MMP-2 and MMP-9 mRNA levels in Caco-2 cells were significantly increased (135–145%) after 6 h incubation with EGF, which was inhibited, in a dose-dependent manner, by both dimers (Fig. 2C). The ECG dimer at 5 μM and the EGCG dimer of 15 μM concentrations, fully suppressed EGF-mediated increased MMP-2/9 mRNA levels. Suggesting an effect at the levels of transcription, in the presence of EGF, both dimers did not affect MMP-2 and MMP-9 mRNA stability (Supplemental Fig. 2, Fig. S2).

**Fig. 3:**
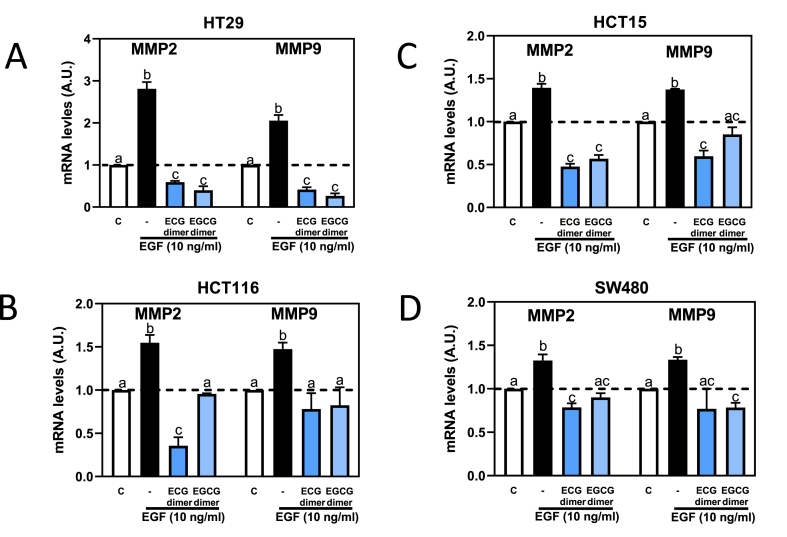
**ECG and EGCG dimers inhibited EGF-mediated MMP-2/9 increased mRNA levels in SW480, HCT15, HCT116 and HT29 cells.** Cells were pre-incubated with or without 5 μM ECG and 15 μM EGCG dimers for 30 min and then with or without EGF (10 ng/ml) for subsequent 6 h. MMP-2/9 mRNA levels were measured by qPCR and referred to actin mRNA levels as housekeeping gene. Results are shown as means ± SEM of 3–5 independent experiments. Values having different superscripts are significantly different (p < 0.05, One-way ANOVA-test).

We next investigated whether ECG and EGCG dimers could mitigate EGF-mediated increases in MMP-2 and MMP-9 mRNA levels in other CRC cell lines. Thus, SW480, HCT15, HCT116, HT29 cells were incubated for 6 h with 5 μM ECG dimer or 15 μM EGCG dimer and in the absence or the presence of EGF. Upon incubation with EGF, all cell lines tested showed an increase in mRNA levels of both MMPs, although at different extents (140–280% over control values) (Fig. 3A–D). Similar to the effects observed in Caco-2 cells, both ECG (5 μM) and EGCG (15 μM) dimers inhibited EGF-mediated increase in MMP-2 and MMP-9 mRNA levels in all the tested CRC cell lines.

The above results suggest that ECG and EGCG dimers could inhibit EGF-induced CRC cell invasion by downregulating MMP-2 and MMP-9 mRNA levels and inhibiting their activity.

### 3.2 ECG and EGCG dimers inhibited EGF-mediated activation of the EGFR and downstream of NF-κB, Akt, and ERK1/2 pathways in Caco-2 cells

**Fig. 4:**
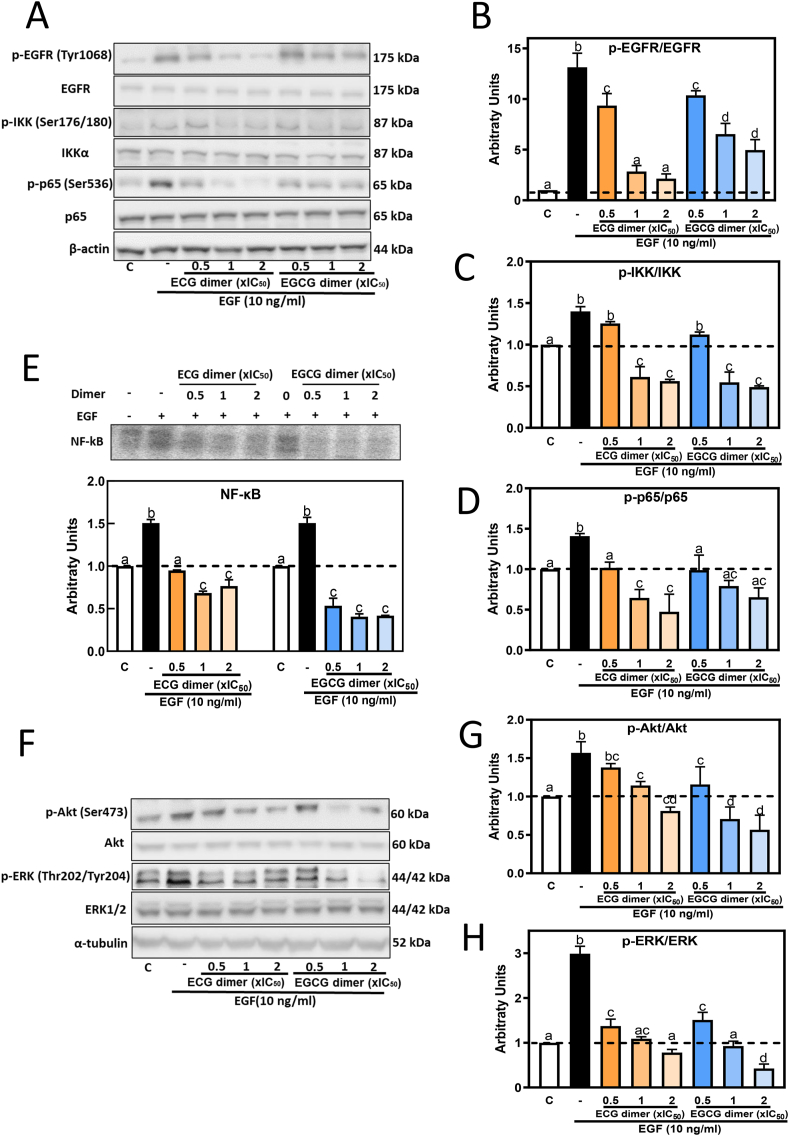
**ECG and EGCG dimers inhibited EGF-mediated activation of the EGFR and downstream of NF-κB, Akt, and ERK1/2 pathways.** Caco-2 cells were pre-incubated with or without ECG and EGCG dimers for 30 min and then with or without EGF (10 ng/ml) for subsequent 10 min. Phosphorylation levels of B- EGFR (Tyr1068), C– IKK (Ser176/180), D- p65 (Ser536), G- Akt (Ser473), and H- ERK1/2 ((Thr202/Tyr204) were evaluated by Western blot. A, F- Representative Western blot images. Bands were quantified and values for phosphorylated proteins were referred to the respective total protein content. E− NF-κB activation was also evaluated by EMSA measuring NF-κB-DNA binding in nuclear fractions. Results are shown as means ± SEM of 3–5 independent experiments. Values having different superscripts are significantly different (p < 0.05, One-way ANOVA-test).

Binding of EGF to the membrane lipid rafts-located EGFR initiates the activation of the receptor and of downstream signals, i.e. Akt, ERK1/2 and NF-κB [24,50,51]. We next investigated the effect of ECG and EGCG dimers on EGF-mediated activation of these signals. In Caco-2 cells and after 10 min incubation, EGF (10 ng/ml) caused a significant increase in the phosphorylation levels of EGFR at Tyr1068, Akt at Ser473, ERK1/2 at Thr202/Tyr204, IKK at Ser176/180, and p65 at Ser536, being IKK and p65 components of the NF-κB pathway. Both ECG and EGCG dimers mitigated EGF-induced increase in the phosphorylation levels of EGFR, IKK, p65, ERK1/2 and Akt in a dose-dependent manner (Fig. 4A, B, C, D, F, G, H). In agreement with the observed increased phosphorylation of IKK and p65, EGF caused a 1.6 folds increase in nuclear NF-κB-DNA binding, as evaluated by EMSA. This was dose-dependently inhibited by ECG and EGCG dimers (Fig. 4E). Given that transcription factor NF-κB binds to the promoter of MMP-2 and MMP-9 genes to regulate their expression [52], its inhibition by the dimers can in part explain their capacity to downregulate these enzymes.

### 3.3 NADPH oxidase inhibitors and ECG and EGCG dimers inhibited EGF-mediated oxidant production and the EGFR signaling pathway in Caco-2 cells

**Fig. 5:**
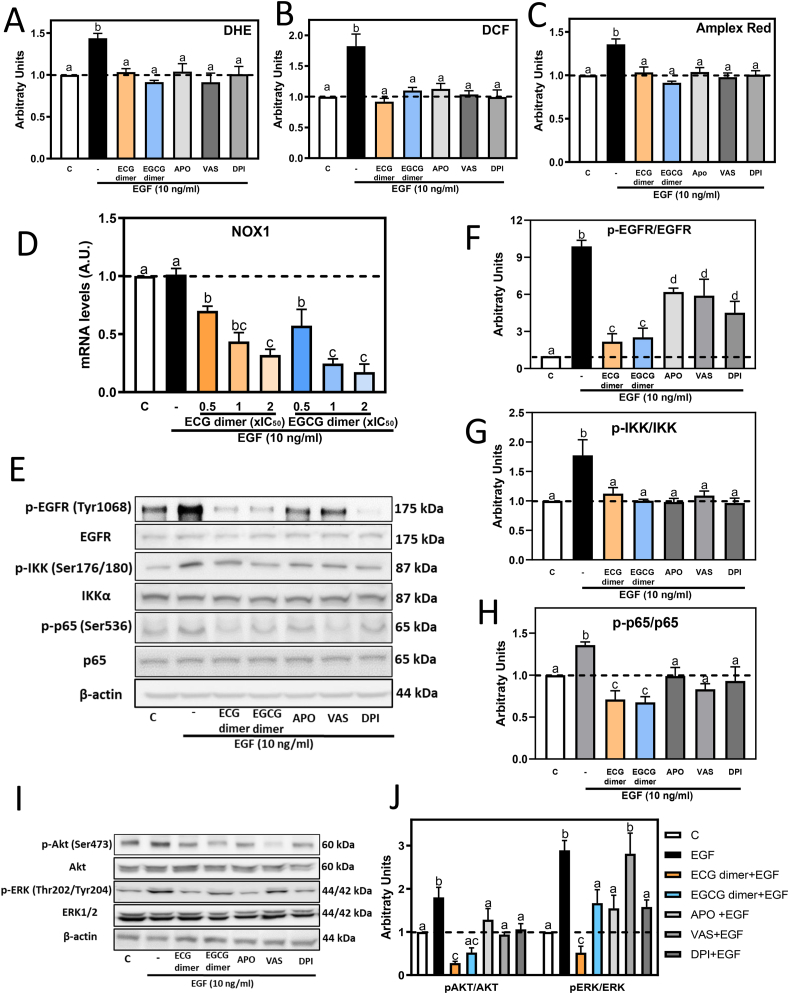
**NADPH oxidase inhibitors and ECG and EGCG dimers inhibited EGF-mediated oxidant production and the EGFR signaling pathway in Caco-2 cells.** Caco-2 cells were pre-incubated with or without 10 μM ECG, 30 μM EGCG, 1 μM apocynin, 1 μM Vas or 1 μM DPI dimers for 30 min and then with or without EGF (10 ng/ml) for subsequent 10 min or 6 h. A-C- Oxidant levels were measured using the probes A- DHE, B- DCF and C- Amplex Red as described in methods. D- NOX1 mRNA levels were measured by qPCR and referred to actin mRNA levels as housekeeping gene. E-J Phosphorylation levels of F- EGFR (Tyr1068), G- IKK (Ser176/180), H-p65 (Ser536), J- Akt (Ser473), and ERK1/2 (Thr202/Tyr204) were evaluated by Western blot. E, I- Representative Western blot images. Bands were quantified and values for phosphorylated proteins were referred to the respective total protein content. Results are shown as means ± SEM of 3–5 independent experiments. Values having different superscripts are significantly different (p < 0.05, One-way ANOVA-test). (For interpretation of the references to color in this figure legend, the reader is referred to the Web version of this article.)

The activation of the EGFR is associated with a rapid activation of cell membrane NOX, which in intestinal epithelial cells is the NOX1 isoform [22]. NOX activation leads to a transient superoxide anion production, a consequent increase in hydrogen peroxide which further activates downstream pathways that cause MMPs upregulation [53]. Thus, we next investigated if ECG and EGCG dimers could modulate EGF-mediated oxidant increase, also characterizing the action of three NOX inhibitors, i.e. Apo, Vas and DPI. This was in part evaluated with the non-fluorescent probes DHE and DHDCF, which cross the cell membrane and fluorescence upon oxidation. After 10 min incubation with EGF, a 48% and 80% increase in DHE and DCF fluorescence was observed, which was fully prevented by preincubating cells with 1xIC_50_ ECG dimer (10 μM) or EGCG dimer (30 μM) or 1 μM NOX inhibitors (Fig. 5A, **B**). H_2_O_2_ concentration in the cell culture medium was measured with the Amplex Red/Peroxidase assay. After 10 min incubation, EGF caused a 40% increase in H_2_O_2_ levels, which was prevented by ECG and EGCG dimers and the three NOX inhibitors (Fig. 5C). The inhibition of EGF-mediated oxidant production by Apo, Vas, and DPI supports that the increase in cell oxidants is dependent on NOX1 activation. Additionally, the rapid oxidant decrease mediated by both dimers suggests that they could directly inhibit NOX1 activity. Supporting such inhibitory action, molecular docking results (Supplemental Fig. 1, Fig. S1) support the potential capacity of the dimers to interact with NOX1.

We next evaluated if NOX1 mRNA levels could be affected by the ECG and EGCG dimers. While incubation with EGF for 6 h did not affect NOX1 mRNA levels, both ECG and EGCG dimers decreased NOX1 mRNA levels in a dose-dependent manner (Fig. 5D). Findings that, in the presence of EGF, both dimers did not affect NOX1 mRNA stability (Supplemental Fig. 2, Fig. S2), suggesting that their capacity to decrease NOX1 mRNA levels occur at the level of transcription. The above results suggest that ECG and EGCG could act both inhibiting NOX1 activity and gene expression.

We next investigated the effects of the three NOX inhibitors Apo, VAS-2870 and DPI on EGF-triggered EGFR, IKK, p65, Akt and ERK1/2 activation. Like that observed for ECG and EGCG dimers, after 10 min incubation with EGF, pre-treatment with the NOX inhibitors for 30 min caused a partial inhibition of EGFR phosphorylation at Tyr1068 and a total or partial inhibition of IKK, p65, ERK1/2 and Akt phosphorylation (Fig. 5E–J). Overall, results support the concept that, the inhibition of NOX1 and consequent decreased O_2_^**.-**^/H_2_O_2_ production, in part contribute to the inhibition by ECG and EGCG dimers of EGF-mediated EGFR signaling pathway activation.

### 3.4 NADPH oxidase inhibitors mitigated EGF-mediated increase in MMP-2/9 mRNA levels in Caco-2 cells

**Fig. 6:**
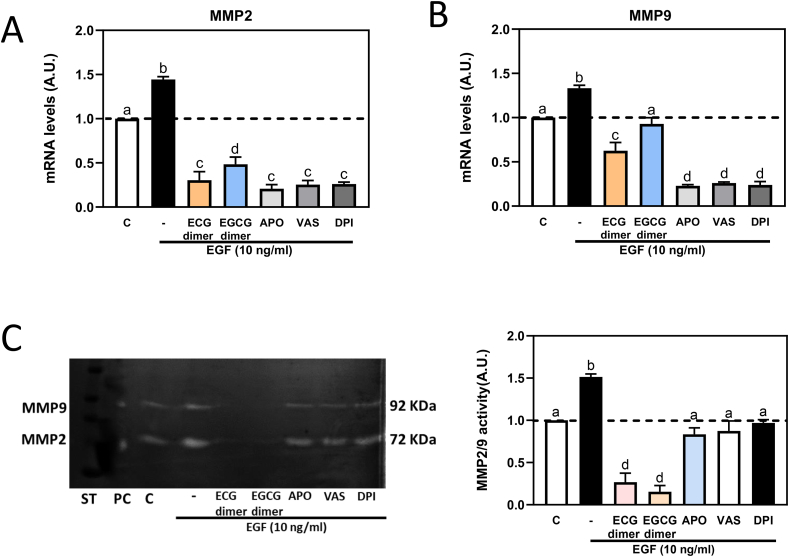
**NADPH oxidase inhibitors mitigated EGF-mediated MMP-2/9 activation and increased MMP-2/9 mRNA levels in Caco-2 cells.** Caco-2 cells were pre-incubated with or without 10 μM ECG, 30 μM EGCG, 1 μM apocynin, 1 μM Vas or 1 μM DPI for 30 min and then with or without EGF (10 ng/ml) for subsequent 6 h. A- MMP-2, B- MMP-9 mRNA levels were measured by qPCR and referred to actin mRNA levels as housekeeping gene. C- MMP-2 and MMP-9 activity was measured by zymography in the cell culture medium. Results are shown as means ± SEM of 3–5 independent experiments. Values having different superscripts are significantly different (p < 0.05, One-way ANOVA-test).

After 6 h incubation, and as observed for the ECG and EGCG dimers, the three NOX inhibitors Apo, VAS-2870 and DPI decreased EGF-mediated increase in MMP-2 and MMP-9 mRNA levels (Fig. 6A–B). Accordingly, both dimers and the NOX inhibitors prevented EGF-triggered MMP-2 and MMP-9 activation (Fig. 6C). Findings that ECG and EGCG inhibit MMP-2/9 activity at a larger extent than the NOX inhibitors, suggest that besides NOX1 downregulation, other mechanisms are involved in the capacity of the dimers to inhibit MMP-2/9 gene expression and/or activity.

### 3.5 NOX1 silencing prevents EGF-mediated increased MMP-2/9 mRNA levels, activation of the EGFR signaling pathways and cell invasion in Caco-2 cells

**Fig. 7:**
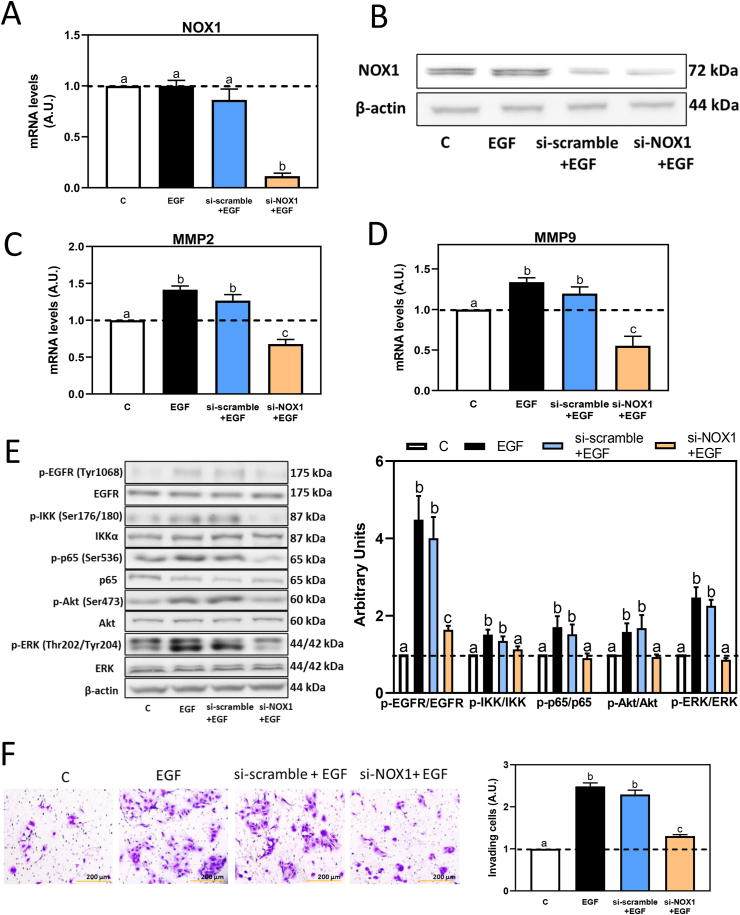
**Silencing of NOX1 inhibited EGF-induced cell invasion, MMP-2 and MMP-9 overexpression, signaling pathways downstream the EGFR in Caco-2 cells, and cell invasion.** Caco-2 cells were transfected with or without scramble (si-scramble) or NOX1 (si-NOX1) silence RNAs, for 48 h, and subsequently incubated with or without EGF (10 ng/ml) for A-D, 6 h, E− 10 min and F-24 h. A- NOX1 mRNA levels and B- NOX1 protein levels were evaluated by PCR and Western blot, respectively. C- MMP-2 and D- MMP-9 mRNA levels were measured by qPCR and referred to actin mRNA levels as housekeeping gene. E− Phosphorylation levels of EGFR (Tyr1068), IKK (Ser176/180), p65 (Ser536), Akt (Ser473) and ERK1/2 (Thr202/Tyr204) were evaluated by Western blot. Bands were quantified and values for phosphorylated proteins were referred to the respective total protein content. (F) Caco-2 cells were transfected with si-scramble or si-NOX1 RNAs, and 48 h later replated into Matrigel chambers. Cells were then stimulated with EGF (10 ng/ml) for 24 h, and cell invasiveness was evaluated (20X magnification). Results are shown as means ± SEM of 3–5 independent experiments. Values having different superscripts are significantly different (p < 0.05, One-way ANOVA-test).

We next assessed the role of NOX1 on EGF-triggered MMP-2/9 upregulation and induction of cell invasion, using NOX1 silencing RNA to knock down NOX1 gene expression in Caco-2 cells, while a scramble silencing RNA was used as a negative control. After 24, 48 and 72 h silencing, NOX1 mRNA levels were decreased by about 20, 85 and 75%, respectively (data not shown). Thus, a 48-h silencing period was selected. Treating cells with EGF for 6 h did not affect NOX1 mRNA level, while NOX1 silencing for 48 h in the absence or the presence of EGF, caused an 85% decrease in mRNA levels and a 35% decrease in NOX1 protein levels compared to the control group (Fig. 7A and B). In cells incubated with EGF for 6 h, scramble silencing caused 10% decrease of MMP-2 and MMP-9 mRNA levels, while NOX1 silencing fully prevented EGF-mediated MMP-2 and MMP-9 mRNA levels increase (Fig. 7C–D). NOX1 silencing also affected cell signaling downstream the EGFR. After 10 min incubation with EGF, scramble silencing did not have a significant effect, but NOX1 silencing caused a total inhibition of EGFR, IKK, p65, ERK1/2 and Akt phosphorylation (Fig. 7E). After incubation with EGF for 24 h, knockdown of NOX1 significantly suppressed Caco-2 cell invasion capacity (Fig. 7F). These results support the involvement of NOX1 on EGF-dependent MMP-2/9 upregulation and Caco-2 cell invasiveness.

## 4 Discussion

We previously described that ECG and EGCG dimers inhibit CRC cell growth and induce apoptosis by inhibiting the EGFR pathway in part through their actions at lipid rafts [39]. The present study investigated the role of NOX1 in CRC cell invasiveness and its involvement in the capacity of ECG and EGCG dimers to inhibit EGF-induced CRC cell invasion. Results show that NOX1 is central to CRC cell invasiveness by amplifying signaling cascades downstream the EGFR, which result in the upregulation of MMP-2 and MMP-9. ECG and EGCG dimers acted inhibiting NOX1 activity and decreasing NOX1 mRNA levels and downstream, EGFR-mediated MMPs expression and cell invasiveness. ECG and EGCG dimers also directly inhibit MMP-2 and MMP-9 activities. Results provide supporting evidence to the potential capacity of dietary procyanidins to mitigate CRC progression.

The EGFR, a member of the RTKs family of receptors, is overexpressed and overactivated in CRC cells. In fact, therapeutic strategies are being developed targeting EGFR for the treatment of metastatic CRC [54]. Through the remodeling of the extracellular matrix, MMP-2 and MMP-9 are involved in tumor invasion and metastasis [5]. Thus, MMP-2 and MMP-9 are overexpressed in metastatic CRC, being considered of prognostic value for poor survival outcome [11]. We observed that several CRC cell lines show an increase in MMP-2 and MMP-9 mRNA levels in response to EGF, being this response of different magnitude in the different cell lines. Both ECG and EGCG dimers inhibited EGF-induced increase in MMP-2 and MMP-9 mRNA levels, not affecting mRNA stability. This inhibition is consistent with the capacity of the dimers to inhibit signaling pathways downstream the EGFR, i.e. NF-κB, ERK1/2 and Akt, that regulate the expression of MMP-2 and MMP-9. In this regard, NF-κB (κB) binding sites are present in the MMP-9 gene promoter [52]. Akt also modulates MMP-9 expression either through the recruitment of p300 to κB sites [55] or by activating NF-κB through IKK phosphorylation [56]. Besides MMPs expression, other site of potential regulation is a direct inhibition of enzyme activity or of the proteolytic cleavage of proenzymes to the active MMP forms. At the lowest concentration tested, both dimers had limited or no effect on MMP-2 and MMP-9 mRNA levels, but fully inhibited MMP-2/9 activity. Polyphenols are capable of binding to proteins/MMPs via their hydroxyl groups (-OH) and galloyl moieties [57,58]. Thus, the interaction of the abundant –OH and galloyl moieties of the dimers with MMP-2/9 proteins released to the cell culture medium, could explain a direct inhibitory action of the dimers on MMP2/9 proteinase activity. Overall, both ECG and EGCG dimers have a dual effect downregulating MMP-2 and MMP-9, one at the transcriptional level, and the other inhibiting MMPs activity and/or processing.

Lipid rafts are sphingolipid and cholesterol-enriched membrane microdomains that provide a platform for the recruitment of receptors and the assembly/activation of NADPH oxidases that act potentiating receptor-mediated cell signaling. NOX1 is the main cell membrane isoform present in enterocytes [59]. Binding of EGF to lipid raft-located EGFR leads to NOX1 activation and to a transient increase in O_2_^.-^ [21,60]. H_2_O_2_ generated from O_2_^.-^ in the extracellular space, crosses the cell membrane and can potentiate EGFR activation through the oxidation of cysteine residues within protein tyrosine phosphatases (PTPs) leading to enzyme inactivation. This decreases PTP-mediated removal of phosphor tyrosine groups prolonging the EGFR cascade [40,61]. Additionally, NOX1-mediated increase of H_2_O_2_ causes oxidation of a Cys797 residue in the EGFR, increasing the receptor’s tyrosine kinase activity leading to EGFR activation [61]. We currently observed that EGF-mediated transient increase in oxidants was prevented by ECG and EGCG dimers and by three different NOX inhibitors. Pointing to the critical role of NOX1 in enhancing the EGFR pathway, dimers and NOX inhibitors also inhibited EGFR Tyr1068 phosphorylation and downstream Akt and ERK1/2 activation. The absence of any detected impact of Vas-2870 on the phosphorylation of ERK1/2 may be attributable to effects of Vas-2870 that are unrelated to the activation of ERK1/2 through the EGFR. Taken together, the above findings suggest that the suppression of NOX1 and the resultant reduction in O_2_^.-^/H_2_O_2_ production represent a key mechanism associated with the inhibition of the EGFR signaling cascades activation by ECG and EGCG dimers. In agreement with previous findings [19,62], this mechanism explains the capacity of ECG and EGCG dimers to mitigate EGF-mediated MMP-2 and MMP-9 increased mRNA levels, and to decrease CRC cell migration and metastasis.

NOX1 is proposed to be involved in the GI tract innate immune responses and in GI carcinogenesis [16], being overexpressed in cancers affecting the large and small intestine [59]. NOX1 promotes proliferation of CRC cells in part by modulating redox-regulated signal transduction [17]. Inhibition of NOX activity with pharmacological pan-NOX inhibitors decreases CRC cancer cell proliferation [63]. *In vivo*, inhibition of host NOX1 blocks CRC tumor growth [64], and small hairpin RNA-mediated NOX1 silencing suppresses tumor growth in mouse models of colon cancer [65]. In support of the central role of NOX1 in CRC invasion and metastasis, upon NOX1 silencing we observed in Caco-2 cells that NOX1 is required for EGF-mediated: i) EGFR pathway activation, ii) upregulation of MMP-2 and MMP-9, and iii) cell invading activity. The ECG and EGCG dimers seem to have a dual effect on NOX1. The observed prevention of the rapid EGF-mediated oxidant increase can be due to a direct inhibition of the enzyme or to changes in lipid raft environment leading to decreased NOX1 activity [66,67]. Our findings through molecular modeling of the interactions between the dimers and NOX1 strongly support a direct inhibitory action of the dimers on NOX1 activity. The C-terminal domain of ***β***-subunits on the membrane of NOX provides the binding site for NADH and FAD. Electrons from NADPH are first transferred to co-enzyme FAD and later to the heme group of the catalytic core, which oxidized O_2_ to O_2_^−^ [68]. ECG and EGCG dimers both could competitively interact with the “FAD binding pocket” of NOX by hydrogen bonds and various hydrophobic interactions. Additionally, both dimers also inhibited NOX1 gene transcription, not affecting NOX1 mRNA stability. This can constitute a longer-term mechanism of downregulation of NOX1 activity. The capacity of the dimers to suppress NOX1 transcription can be due to their actions inhibiting the NF-κB and ERK1/2 pathways, which are both involved in the regulation of NOX1 transcription [69,70]. Overall, while other steps in the EGFR pathway and downstream signals can be regulated by procyanidins [21,39], NOX1 emerges as a central target in ECG and EGCG dimer anti-CRC actions [39].

In summary, the present study provides evidence that ECG and EGCG dimers can inhibit CRC cell invasion by downregulating MMP-2 and MMP-9 both, via NOX1/EGFR-dependent decreased MMPs gene transcription and through a direct effect of the procyanidins on MMPs enzyme activity. We observed that NOX1 plays a central role in enhancing the activation of the EGFR, downstream signaling pathways and MMP-2 and MMP-9 mRNA levels. Thus, the capacity of ECG and EGCG dimers to inhibit NOX1 activity and expression emerge as a central mechanism in their capacity to inhibit CRC cell invasiveness. Considering that the over-activation or over-expression of the EGFR, MMPs and NOX1 are implicated in CRC development and metastasis, the identification of dietary compounds that inactivate them, would help design dietary strategies to mitigate CRC development and progression.

## Declaration of competing interest

Authors have no conflict of interest to declare.

## Data Availability

Data will be made available on request.
