# Supplementary material for: NADPH oxidase 1: A target in the capacity of dimeric ECG and EGCG procyanidins to inhibit colorectal cancer cell invasion
Source: Redox Biol. 2023 Jul 25;65:102827. doi: 10.1016/j.redox.2023.102827 (PMC10410180; doi:10.1016/j.redox.2023.102827)
Supplement: Multimedia component 1 [file mmc1.pdf]

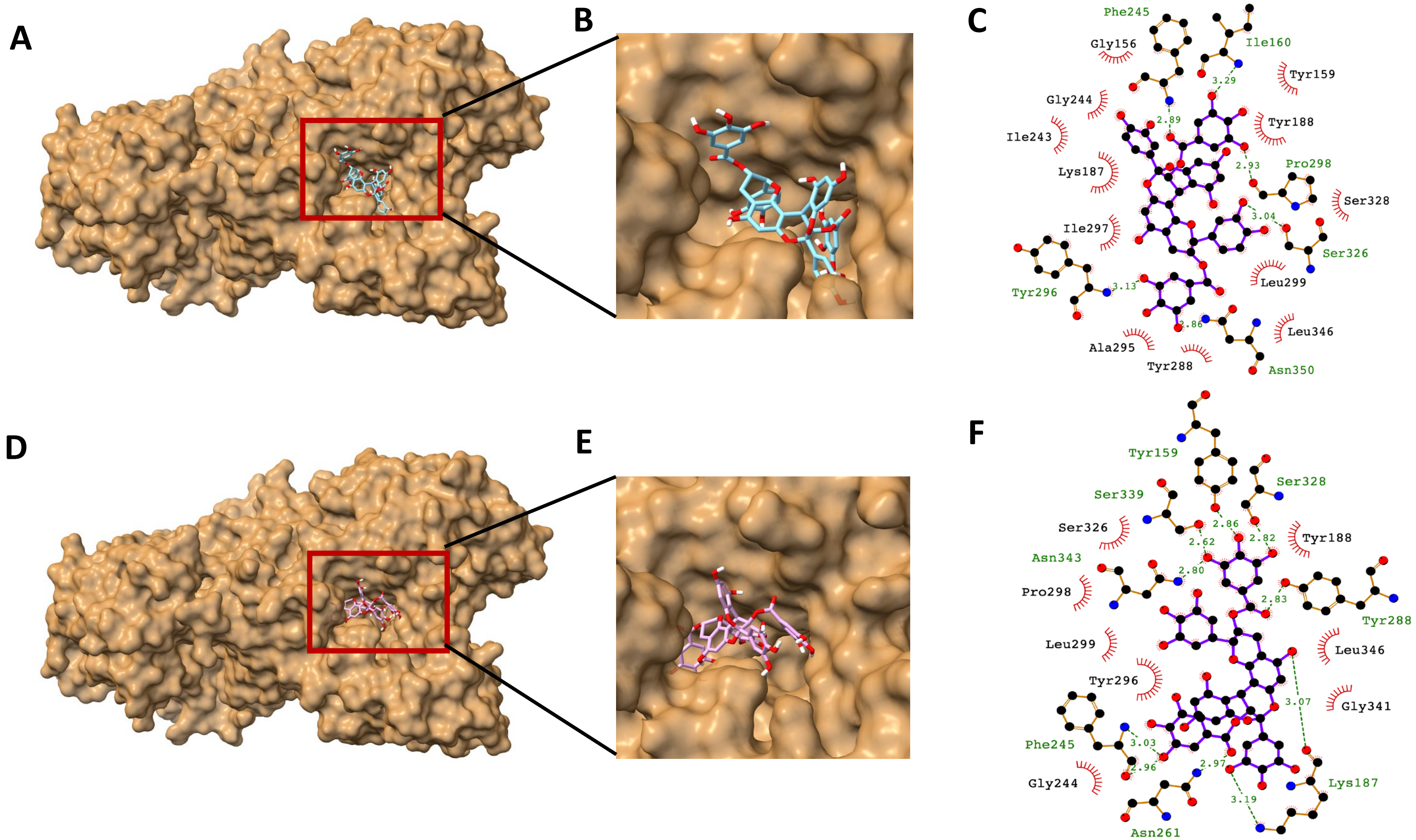

Fig. S1. Potential interactions between ECG dimer (A, B, C), EGCG dimer (D, E, F) and NOX1 were characterized by molecular docking. ECG and EGCG dimers can interact with the FAD binding pocket of NOX1 by hydrogen bonds and various hydrophobic interactions. Panels A, D indicate the position where dimers could bind to the enzyme. Panels B, E show an enlarged view of the binding pocket where dimers could interact with NOX1. Panels C, F show the amino acids in NOX1 that can establish hydrogen bonds (green) and various hydrophobic interactions (red) with the dimers.

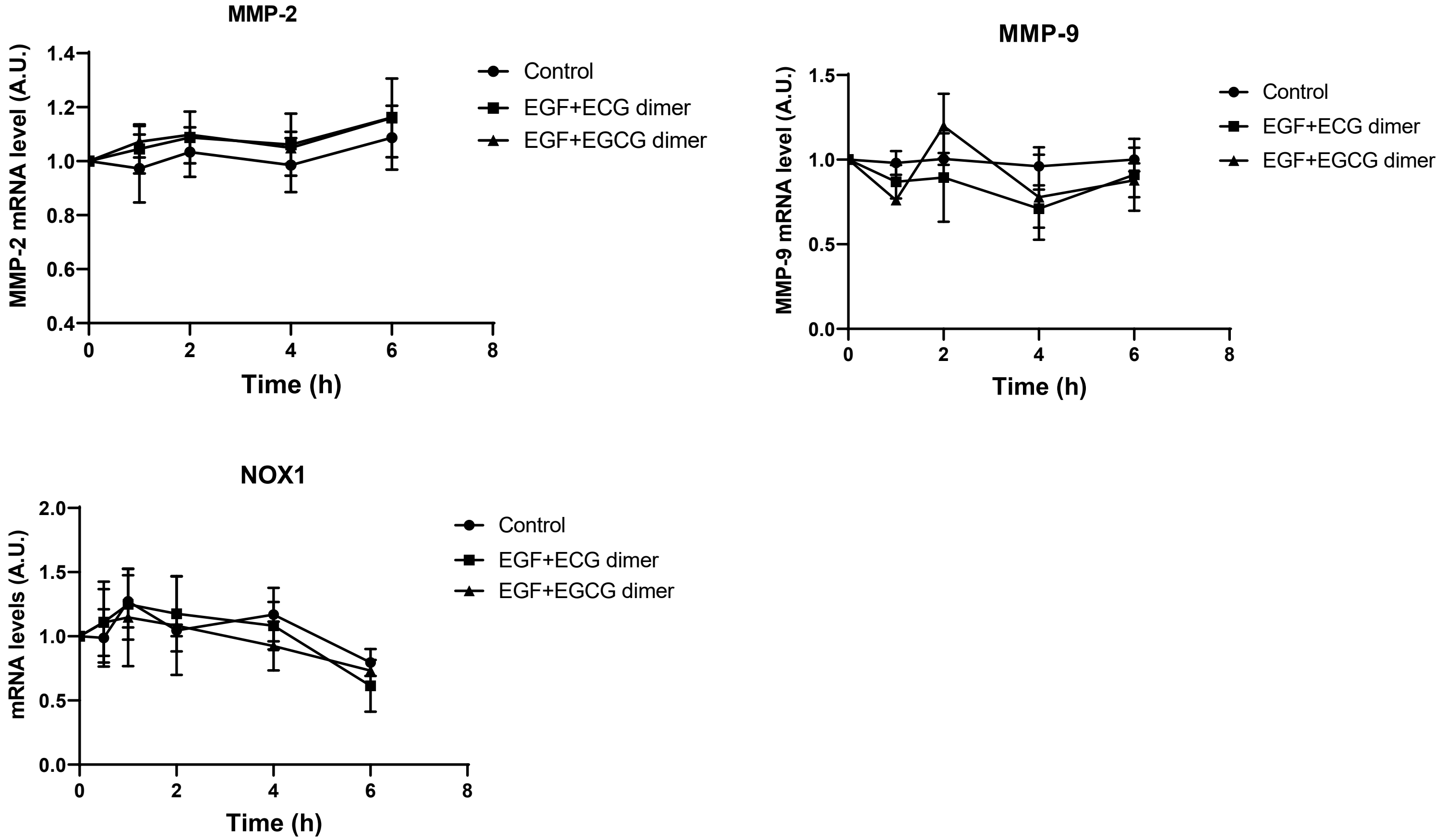

Fig. S2. Effects of EGF and of the ECG and EGCG dimers on MMP-2, MMP-9 and NOX1 mRNA stability. Cells were treated with or without EGF (10 ng/ml) and 10 µM ECG dimer or 30 µM EGCG dimers for 6 h. Then cells were treated by 10 µg/ml actinomycin D to inhibit gene transcription. mRNA was isolated after 0, 0.5, 1, 2, 4, and 6 h incubation, and qPCR analysis was done was done for MMP-2, MMP-9 and NOX1 mRNAs. For the calculation,

we normalized the Ct average value of each time point to the Ct average value at t = 0 to obtain ΔCt values for the control (no additions) and treatment group (ΔCt = (Average Ct of each time point - Average Ct of t=0). To calculate the relative abundance for each time point we calculated $2^{(-\Delta CT)}$. Results are shown as mean ± SEM (n=3).
